# Gel-Type Electrofluorochromic Devices for Advanced Optoelectronic Applications

**DOI:** 10.3390/gels11080673

**Published:** 2025-08-21

**Authors:** Xuecheng Wang, Lijing Wen, Jinxia Ren, Yonghen Wen, Yonghua Li, Yizhou Zhang, Kenneth Yin Zhang

**Affiliations:** 1 State Key Laboratory of Flexible Electronics (LoFE) & Jiangsu Key Laboratory of Smart Biomaterials and Theranostic Technology, Institute of Advanced Materials (IAM), Nanjing University of Posts & Telecommunications, Nanjing 210023, China; 2 Institute of Advanced Materials and Flexible Electronics (IAMFE), School of Chemistry and Materials Science, Nanjing University of Information Science and Technology, Nanjing 210044, China

**Keywords:** electrofluorochromism, gel-based electrofluorochromic devices, gel electrolytes, flexible optoelectronics, smart displays, data encryption

## Abstract

Gel-type electrofluorochromic (EFC) devices, which reversibly modulate photoluminescence under electrical stimuli, have emerged as versatile platforms for advanced optoelectronic applications. By integrating redox-active luminophores with soft, ion-conductive gel matrices, these systems combine the structural robustness of solids with the ionic mobility of liquids, enabling a high-contrast, flexible, and multifunctional operation. This review provides a comprehensive overview of gel-based EFC technologies, outlining fundamental working principles, device architectures, and key performance metrics such as contrast ratio, switching time, and cycling stability. Gel matrices are categorized into ionogels, organogels, and hydrogels, and their physicochemical properties are discussed in relation to EFC device performance. Recent advances are highlighted across applications ranging from flexible displays and rewritable electronic paper to strain and biosensors, data encryption, smart windows, and hybrid energy-interactive systems. Finally, current challenges and emerging strategies are analyzed to guide the design of next-generation adaptive, intelligent, and energy-efficient optoelectronic platforms.

## 1. Introduction

Stimuli-responsive materials have attracted increasing interest in recent years due to their ability to reversibly modulate physical or chemical properties in response to external cues such as thermal changes [1], photoirradiation [2,3], mechanical stress [4,5] or pH changes [6]. Among various external inputs, electrical stimuli are particularly attractive due to their precision, low power consumption, and ease of integration into optoelectronic devices. Electrofluorochromic (EFC) materials represent a promising class which can dynamically alter their emission properties under electrical stimuli [7,8,9]. In contrast to electroluminescent systems, which emit light via charge injection and recombination [10,11], EFC systems modulate pre-existing photoluminescence through redox-driven photophysical processes upon application of an external voltage. This efficient modulation enables low-energy, reversible, and precise spatial control over emission intensity, color, or lifetime, making EFC materials appealing for display technology [12,13], data storage [14], and anti-counterfeiting [15,16].

Numerous EFC systems have been developed by leveraging various luminophores, including conjugated polymers [17,18], fluorescent dyes [19,20], and transition-metal complexes [21,22,23]. These materials operate through the redox-induced modulation of excited-state energy levels or non-radiative decay pathways, commonly involving mechanisms such as photoinduced electron transfer (PeT) and energy transfer [24,25]. However, challenges remain in achieving high contrast, rapid response times, and long-term electrochemical stability in EFC devices. Addressing these limitations requires not only molecular-level design but also the rational engineering of device-level components and architectures. Liquid electrolyte systems or rigid solid-state architectures have been used to develop EFC devices. While liquid systems often exhibit a fast response and high contrast ratios (CR), they suffer from leakage, volatility, and poor mechanical integrity, making them unsuitable for long-term or practical use [26,27]. On the other hand, solid-state devices, though mechanically stable, frequently exhibit a low switching rate due to limited ion mobility and phase separation at the interface [28,29].

Gels are three-dimensional, cross-linked polymer networks capable of retaining large amounts of solvent while maintaining a semi-solid structure. Gel systems often exhibit high softness, excellent pliability and stretchability, self-healing behavior, biocompatibility, and biodegradability [30,31]. Their unique combination of fluid-like ion mobility and solid-like dimensional stability makes them particularly suitable as soft electrolyte matrices in optoelectronic devices [32,33]. A variety of gel-type optoelectronic devices have been developed based on ionogels [34,35], hydrogels [36,37], and organogels [38,39]. In gel-type EFC devices, the functional gel serves as both the electrolyte medium and structural matrix, embedding the electroactive fluorophores into a confined yet diffusible microenvironment. The choice of gel matrix significantly influences the mechanical strength, ionic conductivity, response speed, and long-term stability of the EFC device. Gel-type EFC platforms enable the fabrication of flexible and printable devices, which are attractive for emerging technologies such as wearable electronics and soft displays [40,41]. Moreover, their ability to accommodate multiple electroactive components within a single phase facilitates the construction of multicolor and patternable EFC devices [42].

Despite several reviews covering EFC materials and devices in general [43,44,45], a focused and comparative analysis of gel-type EFC systems is still lacking. Most previous works have addressed molecular design or general device functions without specifically considering the impact of gel electrolytes. In contrast, gel-based EFC systems uniquely integrate redox-modulated fluorescence with the soft-matter mechanics of ion-conductive gels, offering distinct advantages in terms of flexibility, printability, and structural adaptability. As gel-based materials increasingly underpin the development of flexible, stretchable, and multifunctional optoelectronics, a dedicated and systematic summary of their roles in EFC devices has become both timely and necessary. In this review, we present a comprehensive overview of gel-type EFC devices as a distinct and rapidly growing subclass of EFC technologies, emphasizing their working mechanisms, device configurations, and emerging applications. Recent advances in gel-based EFC platforms for applications in flexible displays, electronic paper, sensors, data encryption, and multifunctional systems have been highlighted. Throughout the manuscript, the advantages of gel-based systems have been elaborated in terms of their structural versatility, functional integration, and suitability for advanced optoelectronic applications. By outlining current challenges and future research directions, we aim to inspire innovation at the intersection of electrochemistry, soft materials, and optoelectronic device engineering.

## 2. Fundamentals of Electrofluorochromism

EFC materials are redox-responsive luminophores whose emissions can be reversibly modulated under an applied potential. Compared to electrochromic (EC) systems that regulate optical absorption [46,47], EFC materials enable active emission switching, making them suitable for applications in displays, anti-counterfeiting, and data encryption. The concept dates back to the 1990s, with Lehn’s group first demonstrating a ruthenium complex covalently linked to a quinone unit that enabled voltage-driven luminescence restoration via redox control [48]. Although early systems using metal complexes established the proof of concept, they suffered from low contrast, slow switching, and poor stability [49,50]. The introduction of triphenylamine (TPA) [51,52] and viologen derivatives [53,54] with well-defined redox behavior significantly improved EFC efficiency and reversibility. Since then, a variety of EFC-active species, such as boradiazaindacene [55,56], Tetraphenylethylene (TPE) [57,58], and covalent organic frameworks [59], have been developed.

### 2.1. EFC Mechanism

The reversible luminescence modulation observed in EFC materials primarily arises from redox-regulated, excited-state processes. Among these, PeT is the most widely adopted and extensively studied mechanism. Energy transfer, intrinsically electroactive fluorophores (IEFs), and several emerging switching modes have also been explored to broaden the design space and functionality of EFC systems. These diverse mechanisms extend the design landscape of EFC materials, enabling a finely tunable, multi-level, and spatially resolved emission control beyond traditional PeT-based platforms.

In typical EFC systems, the functional structure comprises an electroactive unit and a luminophore, which are connected either covalently or through supramolecular assembly. The electroactive unit is responsible for undergoing reversible oxidation or reduction under an applied potential, while the fluorophore provides the optical response. PeT refers to the process in which an electron is transferred between a photoexcited fluorophore and a neighboring electron donor or acceptor moiety [60,61]. PeT occurs when a reduced donor transfers an electron to the excited-state fluorophore or when an oxidized acceptor withdraws an electron from it; both scenarios suppress photon emission. Applying a voltage alters the redox state of the quencher, thereby switching the PeT process on or off and restoring or suppressing luminescence accordingly. The efficiency of PeT-driven modulation depends on orbital alignment, driving force, and spatial proximity, making it one of the most widely adopted and tunable strategies in EFC material design [62,63,64,65,66].

**Figure 1:**
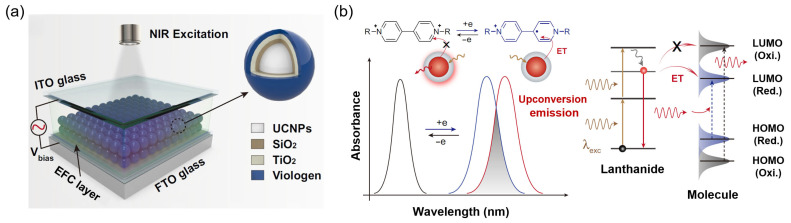
Voltage-controlled FRET-based fluorescence switching in a hybrid EFC device. (**a**) Schematic illustration of the hybrid EFC device based on UCNPs with viologen molecules; (**b**) schematic illustration of electrochemically controlled energy transfer from UCNPs to viologen molecules. Reproduced from Ref. [69]. Licensed under CC BY 4.0.

Beyond PeT, several alternative mechanisms have been employed to achieve voltage-controlled fluorescence switching in EFC systems. Förster resonance energy transfer (FRET) is a long-range dipole–dipole coupling mechanism, wherein the excited-state energy of the donor fluorophore is transferred non-radiatively to an acceptor molecule whose absorption spectrum overlaps with the donor’s emission [67,68]. This process is effective over intermolecular distances of 10–100 Å and is highly sensitive to spectral overlap and spatial orientation. Electrochemical modulation of the acceptor can alter its absorption spectrum, thereby tuning FRET efficiency and enabling voltage-controlled emission. A representative example is provided by Liu and co-workers, who constructed a hybrid EFC device by coupling upconversion nanoparticles, UCNPs, with viologen molecules anchored on their surface (Figure 1a) [69]. Upon the near-infrared (NIR) light excitation, energy transfer from the UCNPs to the reduced viologen species effectively quenched the emission due to spectral overlap, while applying a reverse bias restored luminescence by reversing the redox state of the viologen, thus enabling reversible and dynamic color modulation (Figure 1b).

IEFs refer to luminophores that can undergo reversible redox transformations without the need for external electroactive groups. These redox events directly alter their emissive behavior by influencing conjugation, frontier orbital energies, or excited-state configurations. Dias et al. reported a perylene dipentylimide derivative, which emits at 540 nm in its neutral form [70]. Electrochemical reduction produces radical anion and dianion species that completely quench fluorescence via intramolecular electron transfer. The switching is highly reversible and synchronous with the applied potential. Other IEFs examples include viologen-based polymers undergoing transitions between dicationic and neutral forms [71] and fluoran derivatives exhibiting emission on/off switching via lactone ring opening [72].

Twisted intramolecular charge transfer (TICT) is a redox-gated photophysical mechanism commonly observed in donor–acceptor (D-A)-type molecules with π-conjugated linkers. Upon excitation, the molecule transitions from a planar conformation involving the locally excited state to a twisted D-A conformation, reducing orbital overlap and favoring non-radiative relaxation [73]. This suppresses fluorescence and creates a dark TICT state. Kim and You designed redox-active TICT systems comprising aromatic or antiaromatic donors linked to electroactive acceptors [74]. In their design, applying a reducing potential to the acceptor suppressed the charge-transfer character, restored planarity, and recovered strong fluorescence. The switching was highly reversible and showed good long-term cycling stability.

Electrochemically driven ion migration enables the spatial and reversible control of emissions through the selective accumulation of charged luminophores or ions near device electrodes. Guo et al. reported ion-paired phosphorescent Ir(III) complexes comprising cationic and anionic emitters [75]. Under an applied electric field, the ions migrated to opposite sides of the device, producing a red emission near the anode and green near the cathode. This allowed dynamic emission control and even white-light generation through stoichiometric tuning.

### 2.2. EFC Devices

EFC devices are typically composed of two conductive substrates like ITO-coated glasses or flexible substrates, separated by an active layer containing EFC materials and an electrolyte. Based on how the active layers are integrated, EFC devices are commonly categorized into multilayer and single-layer configurations.

**Figure 2:**
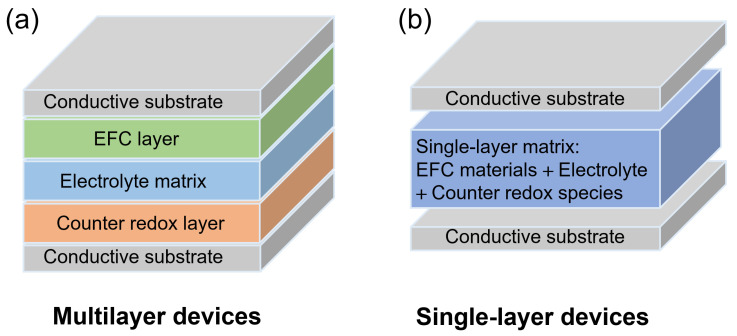
Schematic illustration of multilayer EFC devices (**a**) and single-layer EFC devices (**b**).

Multilayer devices comprise two separate electroactive layers deposited on opposing substrates, with one layer containing the EFC fluorophore and the other hosting a complementary redox component (Figure 2a). Between them lies a medium (liquid, gel, or solid electrolyte) that enables ion transport and electrical insulation. The multilayer approach allows for the spatially discrete optimization of redox behavior, emission wavelength, and switching kinetics. For example, one can independently tune the oxidation potential of the anode component while engineering the emission properties of the cathode fluorophore [76]. However, the multilayer configuration also presents fabrication challenges, such as the need for uniform thin-film deposition, precise layer thickness control, and tight alignment between electrodes. Despite this, such devices are frequently used in mechanistic studies due to their modularity and well-defined interfaces, which facilitate the clearer interpretation of electrochemical and photophysical behavior.

In contrast, single-layer devices integrate all functional components, including EFC material, counter redox species, electrolyte salt, and plasticizer, into a single composite film (Figure 2b). These are typically fabricated via solution casting, drop-coating, and lamination [77,78]. Some systems also employ polymer backbones functionalized with electroactive fluorophores to form single-layer EFC composite films [79]. This configuration dramatically simplifies processing, reduces material waste, and is well-suited for large-area, flexible, and low-cost device fabrication. Furthermore, the mixing of redox pairs within a single matrix often improves ionic conductivity and minimizes resistance. However, single-layer systems may suffer from limited control over component distribution, redox competition, or phase separation, all of which can affect long-term stability.

The integration of EFC materials in either multilayer or single-layer devices requires interface engineering strategies that align with their redox-driven luminescence behavior. Efficient electron or hole injection is necessary to support redox switching, and the choice of electrode material can influence both charge transport and optical transparency. Overall, the structural configuration of EFC devices plays a critical role in determining their photophysical behavior, response speed, and long-term stability.

### 2.3. Performance Parameters of EFC Device

The evaluation of EFC device performance requires a set of standardized, quantitative indicators that reflect both the optical modulation ability and electrochemical response of the system. CR is a fundamental descriptor, defined as the ratio of photoluminescence intensity between the emission “on” (*I_ON_*) and “off” (*I_OFF_*) states at the peak emission wavelength (*λ*_max_) (Equation (1)).

(1) $$\mathrm{CR}=\frac{I_{ON}}{I_{OFF}},$$

Alternatively, the ratio of integrated area (CR_Δ*λ*_) between the emission “on” (*A_ON_*) and “off” (*A_OFF_*) states over the full emission spectrum may be used for broader comparisons (Equation (2)).

(2) $$\mathrm{CR}\Delta \lambda \;=\frac{A_{ON}}{A_{OFF}},$$

Materials or devices with a higher CR are generally more suitable for display applications. However, high CR values often come at the cost of slower switching kinetics or diminished reversibility, requiring careful trade-offs during molecular and device design.

Emission Modulation Depth (ΔFL%) provides a percentage-based evaluation of switching efficacy. This value offers an intuitive measure of optical visibility for applications requiring high contrast under ambient conditions. ΔFL% is calculated according to Equation (3).

(3) $$\Delta \mathrm{FL}\%=\frac{I_{ON}-I_{OFF}}{I_{ON}}\;\times \;100=(1-\frac{1}{CR})\;\times \;100$$

Switching time is a key performance parameter in EFC devices, typically defined as the time required to reach 90% of the fluorescence change after a voltage step. It is governed by factors such as charge transport, film thickness, redox kinetics, and ion mobility. Balancing the CR and switching speed remains a major design challenge. A representative example is provided by Yu et al., who reported a hyperbranched polyimide (HB-TPE-PI) featuring an aggregation-induced emission (AIE) and cavity-rich architecture, which significantly promoted ion diffusion and charge transfer [80]. As a result, the device achieved a remarkable fluorescence switching time of 1.8 s (on) and 0.2 s (off), along with a high CR of 425 and low operation voltage (0–0.25 V), demonstrating the feasibility of ultrafast EFC switching through rational polymer and structural design.

Cycling stability refers to the number of switching cycles an EFC device can undergo without significant performance loss (<10% drop in CR or ΔFL%). Enhancing the stability requires redox-stable luminophores, robust electrolytes, and effective encapsulation against moisture and oxygen. For example, a high-performance gel-type EFC system based on a polyamide incorporating *p*-phenylenediamine and asymmetrical spirobifluorene/TPA units exhibited outstanding cycling stability, retaining a nearly unchanged fluorescence contrast after 200 switching cycles [81]. This performance was attributed to the excellent electrochemical stability of the redox-active units and the minimized intermolecular π–π interactions afforded by the bulky, twisted molecular design.

Currently, there is a lack of established mathematical models that quantitatively describe the structure–performance relationships in EFC devices. In contrast, EC materials and EC devices have benefitted from various modeling approaches that correlate intrinsic material properties with device-level performance metrics [82]. Such frameworks could be adapted to the EFC field to enable predictive optimization prior to fabrication. For instance, the fluorescence switching time (τ) may be approximated by a diffusion-limited relation (Equation (4)), where L is the electroactive layer thickness and D is the ion diffusion coefficient in the gel electrolyte, obtainable from electrochemical impedance spectroscopy or predicted via molecular dynamics (MD) and density functional theory (DFT) simulations.

(4) $$\tau \;\approx \;\frac{L^{2}}{D}$$

Similarly, CR and ΔFL% can be linked to changes in the optical band gap (ΔEg) and oscillator strength, which are accessible through time-dependent DFT (TDDFT) calculations. Cycling stability may be evaluated by simulating redox potential windows, molecular orbital distributions, and reorganization energies to anticipate degradation pathways. Incorporating such modeling strategies, adapted from EC research, could bridge the current gap in the EFC field and accelerate the rational design of gel electrolytes and electroactive architectures for advanced optoelectronic applications.

## 3. Gel Matrices for EFC Devices

EFC devices demand a balance between mechanical integrity, ionic conductivity, and compatibility with electroactive molecules. In this context, gel matrices have emerged as versatile platforms that bridge the gap between liquid electrolytes and rigid solids. These soft, semi-solid materials consist of polymeric or supramolecular networks that entrap liquid components to form stable systems. Depending on the type of retained solvent, gel electrolytes used in EFC devices can be broadly classified into ionogels, organogels, and hydrogels.

### 3.1. Ionogels

Ionogels are a class of solid-like materials formed by confining ionic liquids (ILs) within polymer networks [83]. ILs, typically defined by their low melting points (<100 °C), are molten salts composed of bulky, asymmetric organic cations paired with inorganic or organic anions. They exhibit high ionic conductivity, wide electrochemical windows, low vapor pressure, and outstanding thermal and chemical stability [84,85]. These features make ILs ideal electrolytic media, especially under anhydrous or high-temperature conditions. Ionogels retain the favorable ion transport characteristics of ILs while acquiring mechanical integrity and processability by immobilizing ILs within a solid matrix. Depending on the matrix design, ionogels can exhibit tailored viscoelasticity, thermal resistance, and dielectric behavior, offering tunable platforms for diverse optoelectronic applications.

In EFC devices, ionogels serve as multifunctional solid-state electrolytes that significantly enhance performance. Their intrinsic ionic conductivity ensures rapid charge transport, which is essential for efficient and reversible redox-driven emission modulation. Ionogels can be easily co-processed into uniform composite layers with EFC materials. Viologen-based EFC materials embedded in ionogel systems have been utilized to construct bistable fluorescent displays [86]. Compared to liquid electrolytes, ionogels eliminate leakage and evaporation issues, improving long-term device stability. Meanwhile, the electrochemical inertness and broad voltage tolerance of ILs permit EFC devices to function across a wide electrochemical window without side reactions or photobleaching, paving the way for the integration of diverse EFC components with varying redox thresholds. Moreover, the soft-solid nature of ionogels supports flexible, transparent, and even stretchable EFC device configurations. Recent advances in polymerizable ionic liquid-based ionogels have further expanded the design landscape, offering crosslinked architectures that combine structural robustness with a high ionic mobility [41]. These systems have demonstrated excellent cycling stability, low-voltage actuation, and sustained optical contrast in prototype devices, underscoring their potential for soft optoelectronic technologies.

### 3.2. Organogels

Organogels are formed by the self-assembly of low-molecular-weight gelators or polymeric frameworks in organic solvents [38]. These gels construct a viscoelastic network stabilized by non-covalent interactions such as hydrogen bonding, π-π stacking, or van der Waals forces. Organogels offer a unique advantage in EFC devices by providing a tailored microenvironment. For instance, several EFC molecules showing an aggregation-induced emission exhibited enhanced brightness within the confined space of the organogel matrix due to the restriction of intramolecular motion [87]. In another example, the organic solvent environment within the gel also allows for the modulation of excited-state processes such as TICT [88]. Moreover, organogels offer exceptional solubilizing capabilities for poorly soluble EFC molecules. This solvent-mediated dissolution ensures a homogeneous dispersion within the gel network, effectively preventing phase separation or delamination from the electrode, which is critical for long-term device stability. Consequently, organogel matrices can accommodate a broader range of EFC materials, including those that might otherwise precipitate or aggregate in conventional electrolytes.

### 3.3. Hydrogels

Hydrogels represent a class of three-dimensional polymer networks that can incorporate large amounts of water while maintaining structural integrity [89]. These hydrophilic matrices are typically formed through either the physical entanglement or covalent crosslinking of polymers such as poly(vinyl alcohol) (PVA), polyacrylamide, or natural biopolymers including alginate and gelatin. Their unique architecture combines a high water content with remarkable mechanical compliance, creating materials that exhibit tissue-like elasticity combined with permeability to ions and small molecules. The biocompatibility and tunable physicochemical characteristics of hydrogels have led to their widespread adoption in biomedical applications, ranging from tissue engineering scaffolds to wearable biosensors.

The properties of hydrogels make them particularly suitable for EFC devices. The demonstration of a hydrogel-type EFC device was reported by Adams et al., who developed a multifunctional system incorporating thiazolothiazole (TTz) derivatives within a hydrogel matrix [90]. This system achieved a stable performance over 250 redox cycles, highlighting the protective role of the hydrogel matrix against electrochemical degradation pathways common in liquid electrolyte systems. Such examples highlight the synergy of hydrogel networks with tailored fluorophores and network architectures, allowing for the optimization of switching speed, optical contrast, and cycling stability, making hydrogel-based EFC systems ideal for high-performance applications.

While ionogels, organogels, and hydrogels each offer distinct advantages for EFC applications, their suitability depends on the specific performance demands. Ionogels stand out in terms of ionic conductivity, electrochemical stability, and compatibility with flexible configurations, making them ideal for low-voltage, high-durability devices. Organogels provide excellent solubilization for hydrophobic or poorly soluble fluorophores and support microenvironment-sensitive photophysical processes such as AIE and TICT. In contrast, hydrogels offer a superior biocompatibility and are especially suitable for wearable, soft, or bio-integrated EFC systems. However, their high water content may limit long-term stability under open-air or high-voltage conditions. A comparative understanding of these matrices is essential for the rational design of gel-type EFC platforms tailored to specific optoelectronic applications.

## 4. Applications of Gel-Type EFC Devices

Gel-type EFC platforms combine the mechanical stability of solid-state materials with the efficient ionic transport of liquids, resulting in systems that are leakage-resistant, flexible, and structurally robust. Gel-type EFC devices are being actively explored across a variety of application scenarios including dynamic display panels, optical data security, electronic paper, sensors, and multifunctional systems. The following sections outline key advances in these diverse applications.

### 4.1. Displays

**Figure 3:**
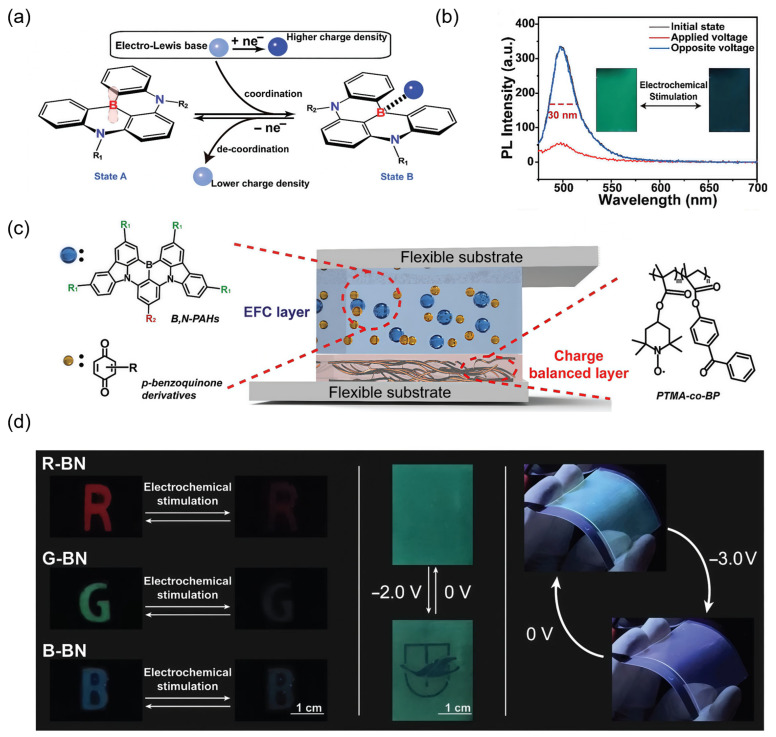
High-performance, multicolor gel-type EFC display based on B,N-PAHs. (**a**) Schematic diagram of the indirect redox strategy of EFC systems based on B,N-PAHs; (**b**) emission spectra of the EFC systems based on B,N-PAHs at different voltages. Inset pictures: Stable and repeatable fluorescence switching upon electrochemical stimulation; (**c**) schematic illustration of the organogel-type EFC device based on B,N-PAHs; and (**d**) the prototypes for a three-primary display, patterned display, and flexible display (*λ*_ex_ = 365 nm). Reproduced from Ref. [42]. Licensed under CC BY 4.0.

To meet the demands of advanced display technologies, particularly in terms of color purity, response speed, and operational stability, Zhang and co-workers developed a gel-type EFC system based on boron–nitrogen embedded polyaromatics, B,N-PAHs, integrated with electrochemically switchable coordination (Table 1) [42]. Inspired by the dynamic boron coordination in biological systems, the authors introduced a reversible redox-controlled interaction between sp^2^-hybridized boron centers and reduced *p*-benzoquinone (*p*-BQ) derivatives, enabling the indirect modulation of luminescence via a reversible sp^2^-to-sp^3^ hybridization of the boron center (Figure 3a). This biomimetic coordination strategy avoided the irreversible degradation typically seen in direct redox systems and allowed stable and repeatable fluorescence switching (Figure 3b). To fully harness the advantages of soft material engineering, a two-layer gel-type EFC device was fabricated using an organogel based on Poly(methyl methacrylate) (PMMA) with tetrahydrofuran as an organic solvent, containing B,N-PAHs and *p*-BQ, paired with a photo-crosslinked PTMA-co-BP ion-storage layer (Figure 3c). This configuration demonstrated a superior performance compared with single-layer and semi-solid analogs, achieving a narrow emission bandwidth (full width at half-maximum = 30 nm), high quenching efficiency (>90%), and fast switching times (*t*_on_ = 0.6 s, *t*_off_ = 2.4 s). Notably, the system supported full-color operation with red, green, and blue EFC units realized through structural tuning of the B,N-PAHs, enabling a high-purity, multicolor display (Figure 3d). The gel matrix not only enhanced ionic mobility and intermolecular coordination but also endowed the devices with excellent mechanical flexibility, enabling reliable fluorescence modulation even under bending conditions (Figure 3d). These features highlight the critical role of gel-type architectures in advancing the practicality, visual performance, and adaptability of EFC-based display technologies.

**Table 1:** Representative gel-based EFC devices and their applications.

EFC Materials	Gel Type	Application	Ref.
B,N-PAHs/p-BQ	Organogel	Displays	[42]
Ir(III) complexes with pyridinium units	Ionogel	Displays	[91,92,93]
EFIL-TTM	Organogel	Displays	[94]
Fc-TPE	Hydrogel	Displays	[95]
Viologen-substituted Ir(III) complexes	Ionogel	Data encryption	[86]
7-Hydroxycoumarin/rhodol/2-(2-(4-hydroxystyryl)-6-methyl-4H-pyran-4-ylidene)malononitrile	Organogel	Data encryption	[96]
TPA segments with AIE cores	Organogel	Data encryption	[97]
AIE-active 9,10-azaboraphenanthrene (BNP) units	Organogel	Data encryption	[98]
Ir(III) complexes with N-H units	Ionogel	Electronic paper	[99,100]
Ir(III) complexes with O-H units	Ionogel	Electronic paper	[101]
Transition-metal complexes with pyridinium units	Ionogel	Electronic paper	[102]
OCBs	Hydrogel	Electronic paper	[103]
[ImTV][TFSI]	Ionogel	Sensors	[41]
BQ/SPM	Organogel	Sensors	[40]
EEIL	Ionogel	Smart windows/Displays	[104]
Tetrabenzofluorene derivatives	Organogel	Smart windows/Displays	[105]
Thienoviologens	Organogel	Smart windows/Displays	[106]
TTz derivatives	Hydrogel	EC/EFC hybrid systems	[90]
PolyZn	Organogel	EC/EFC hybrid systems	[107]
PPAOF/SRB	Organogel	Thermo-responsive EFC device	[108]
Poly(NIPAM-co-TV)	Organogel	Thermo-responsive EFC device	[109]
LY-POMs/Rh6G	Organogel	Energy-interactive EFC device	[110]
TPA-bpy	Organogel	Energy-interactive EFC device	[111]

Zhao and co-workers developed a series of phosphorescent Ir(III) complex-based ionogel systems that enable precise and reversible luminescence control through electrochemical stimuli (Table 1) [91,92,93]. The team systematically introduced viologen-like mono- or bis-pyridinium units into the ligands of Ir(III) complexes to construct a series of redox-responsive luminophores with tunable emissive properties. The redox-triggered emission modulation arises from geometric transformations at the ligand level, which directly impacts emission intensity and color. These complexes were embedded into ionogel-based EFC devices, using 1-butyl-3-methylimidazolium hexafluorophosphate as the ionic medium, and doped with *^n^*Bu_4_NPF_6_ to enhance conductivity. The quasi-solid ionogel matrix served not only as the electrolyte but also as a mechanically stable, leakage-free platform that enabled efficient ion transport and redox switching. Importantly, the Ir(III) complexes bearing hexafluorophosphate counterions exhibited excellent solubility in the BMIM-PF_6_-based ionogel, eliminating the need for additional solvents and ensuring the homogeneous dispersion of electroactive components within the gel phase. These devices exhibited good EFC performance, with a low driving voltage of below 2.5 V, good response times, and high durability under ambient conditions. Among these studies, a representative example featured a flexible gel-type EFC device, in which the electroactive layer was screen-printed onto ITO-coated PDMS substrates. This device showed stable multi-state emission switching under mechanical bending, demonstrating application potential in flexible multicolor displays [92].

**Figure 4:**
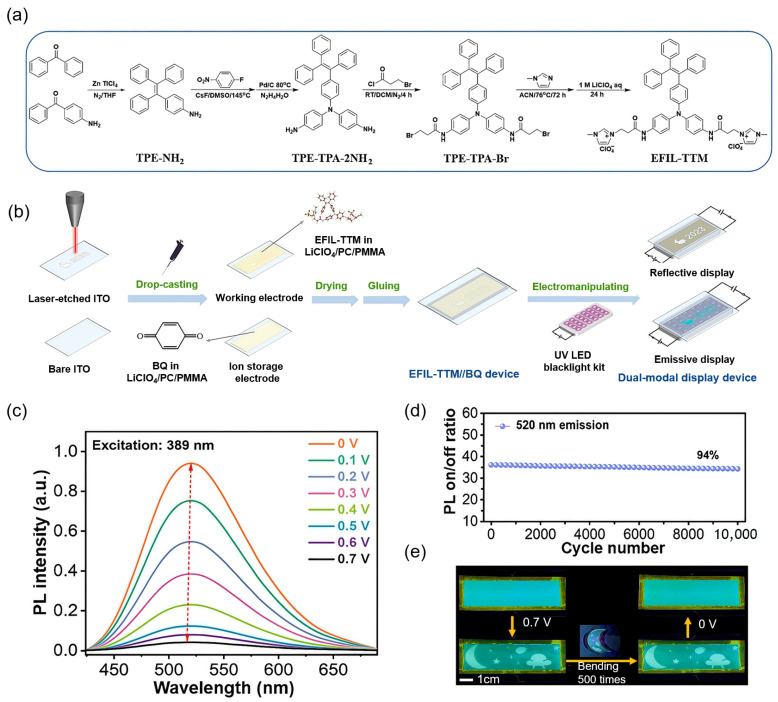
Gel-type EFC device with high durability based on electroactive fluorescent ionic liquid EFIL-TTM. (**a**) The synthesis route of EFIL-TTM; (**b**) schematic illustration of the fabrication of EFIL-TTM EFC device; (**c**) fluorescence emission of the device upon diverse voltages with a retention time of 100 s under the excitation of 389 nm; (**d**) fluorescence on/off ratio after 10,000 cycles with a retention time of 5 s; and (**e**) the emissive display of the flexible EFIL-TTM device before and after 500 times of bending–unbending cycles. Reproduced with permission from Ref. [94]. Copyright © 2023 Elsevier B.V.

In another study, Chao and co-workers reported a gel-type EFC device based on a newly synthesized electroactive fluorescent ionic liquid, EFIL-TTM, which combined a TPA electroactive core, a TPE fluorophore, and imidazolium ionic conductive groups into a single molecule (Figure 4a) [94]. This design not only integrated redox and fluorescent functionalities at the molecular level but also endowed the molecule with AIE and high ionic conductivity, making it ideal for quasi-solid-state EFC systems. The resultant gel-type device utilized a PMMA-based organogel containing acetonitrile as both the electrolyte matrix and the mechanical scaffold, forming a dual-layer configuration with BQ ion-storage gel as the counter electrode (Figure 4b). The gel-type device exhibited outstanding luminescence on–off switching with a low driving voltage of 0.7 V (Figure 4c). It also exhibits a fast response time (*t*_off_ = 1.7 s, *t*_on_ = 2.3 s) and remarkable stability over 10,000 cycles (fluorescence retention > 94%) (Figure 4d). These achievements were attributed to the highly conjugated architecture and ionic nature of EFIL-TTM, which facilitated fast redox transitions and minimized interfacial resistance. Notably, the device also enabled a salt-free operation, leveraging the intrinsic ionic conductivity of EFIL-TTM itself, and supported flexible patterned displays on ITO/Polyethylene terephthalate (PET) substrates with excellent mechanical durability over repeated bending (Figure 4e).

Hydrogels have also been developed as matrices for gel-type EFC devices targeting display applications. Zhang et al. designed a gel-type EFC platform, Fc-TPE, based on a ferrocene–tetraphenylethene conjugate, combining electroactive and AIE features into a single molecular framework (Table 1) [95]. The ferrocene moiety functioned as a redox-active quencher, enabling fluorescence modulation via a PeT mechanism. Upon oxidation of the Fc unit, PeT was suppressed, and a strong blue-white emission was restored, while the reduction in the oxidized species reversed this effect. The resulting molecule was incorporated into a PVA hydrogel matrix along with the ionic liquid [EMIM]BF_4_ and NaCl to construct a gel-type EFC device. The PVA/Fc-TPE hydrogel demonstrated a remarkable EFC performance under low voltage operation (0–1 V), with over 800-fold fluorescence enhancement upon oxidation. Furthermore, the introduction of phytic acid PA into the hydrogel system significantly enhanced its mechanical robustness by strengthening intermolecular hydrogen bonding and suppressing excessive crystallinity. The tensile strength of the PA-doped hydrogels increased from 271 kPa to 351 kPa, with elongation at the break improved from 250% to 350%, while retaining full EFC functionality. Customized gel geometries and 5 × 5 pixelated EFC arrays were fabricated, capable of displaying dynamic patterns and encrypted binary codes. These results underscore the versatility of hydrogel-type EFC platforms based on AIEgens, offering low-voltage operation, flexibility, structural tunability, and smart information display capabilities.

### 4.2. Data Encryption and Anti-Counterfeiting

**Figure 5:**
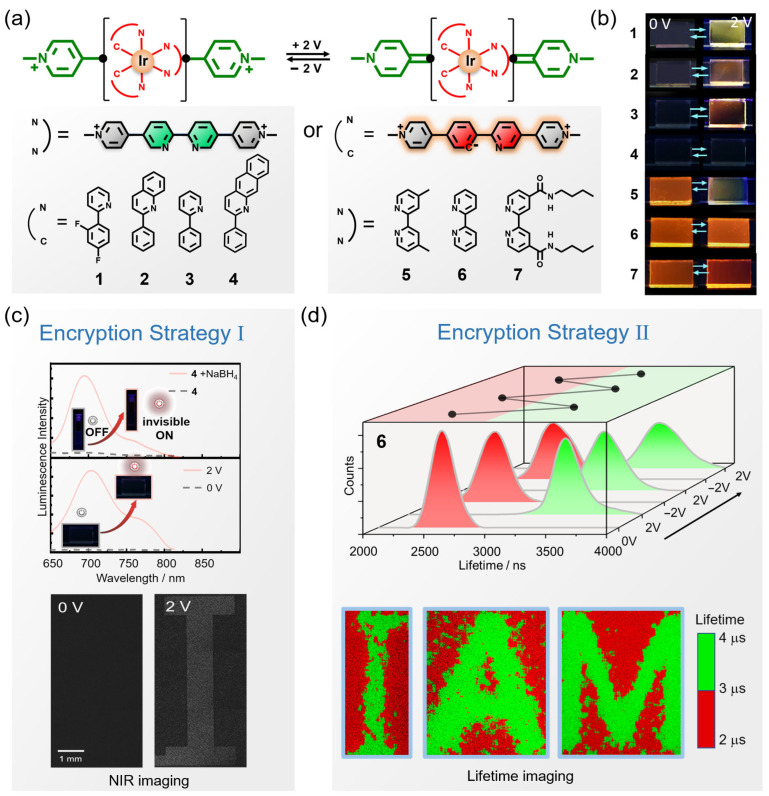
Multidimensional optical encryption strategies enabled by gel-type EFC devices incorporating viologen-substituted Ir(III) complexes. (**a**) Chemical structures and electrochemical conversion of viologen-substituted complexes **1**–**7**; (**b**) photographs of EFC devices containing **1**–**7** toward electrical stimuli under illumination at 365 nm using a handheld UV lamp; (**c**) photoluminescence spectra traces of complex **4** towards chemical reduction and electrical stimulation, and luminescence images of an EFC device with etched information obtained via confocal microscopy before and after electrical stimulation (*λ*_ex_ = 405 nm, *λ*_em_ = 700 ± 50 nm); and (**d**) luminescence lifetime distribution analysis for the lifetime images of complex **6** with the alternating voltages of 2 V and –2 V, and photoluminescence lifetime images of the devices with etched information (*λ*_ex_ = 405 nm, gated time = 3 μs). Reproduced with permission from Ref. [86]. Copyright © 2021 Wiley-VCH GmbH.

The integration of gel-type EFC platforms into data encryption and anti-counterfeiting technologies has attracted increasing attention owing to their capability for multidimensional and stimulus-responsive emission modulation. A representative study by Zhao and co-workers developed a series of viologen-substituted Ir(III) complexes **1**–**7** with tunable EFC behavior via rational ligand engineering (Figure 5a) [86]. The complexes were embedded into quasi-solid 1-butyl-3-methylimidazolium hexafluorophosphate ionogel matrices to fabricate sandwich-type EFC devices that remained transparent. By precisely modulating the electronic communication between the viologen moieties and the luminophore, the authors realized there was not only redox-active on/off luminescence switching (**1**–**4**) but also dual-emissive states with distinct wavelengths and lifetimes (**5**–**7**) (Figure 5b). This study also demonstrated two advanced encryption strategies that significantly enhance the information encryption and functional complexity of gel-type EFC platforms. The first strategy exploited the near-infrared (NIR) emission of the Ir(III) complex **4**, which rendered etched patterns invisible under standard UV inspection yet detectable using specialized NIR imaging systems (Figure 5c). This approach takes advantage of the intrinsic low visibility of NIR light to human eyes, enabling covert tagging and dual authentication, where visible and invisible messages can coexist in the same physical location but be selectively decoded based on spectral access (Figure 5c). The second strategy involved the lifetime-based optical encryption, in which electrochemical stimulation dynamically modulated the photoluminescence lifetime of complex **6** without altering its emission color or intensity (Figure 5d). This device allows for the creation of time-gated etched information that are imperceptible to conventional detectors but can be revealed through time-resolved luminescence imaging. Such lifetime encoding offers an additional security dimension beyond color and brightness and is particularly suitable for high-level anti-counterfeiting applications. This work showcases the potential of gel-based EFC systems for constructing multi-channel and secure optical data encryption platforms.

**Figure 6:**
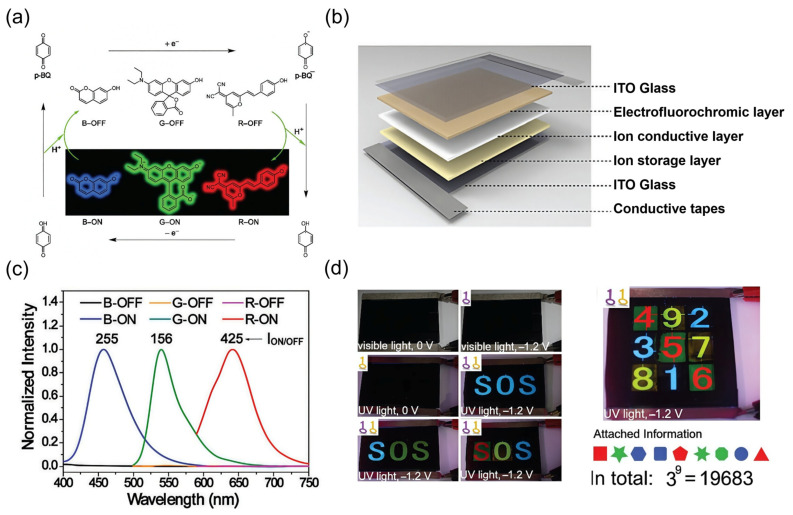
Electro-base-activated RGB gel-type EFC devices for letter and numerical pattern encryption. (**a**) Chemical structures and mechanism of EFC molecules in the RGB color-tunable “on-off” device; (**b**) device architecture showing the sandwich-type configuration of the RGB gel-type EFC system, comprising an EFC emissive layer, PMMA-based ion-conductive gel, and a p-BQ ion-storage layer; (**c**) the fluorescence spectra of the RGB EFC devices, respectively, excited at 380 nm, 480 nm, and 570 nm; and (**d**) the RGB color-tunable EFC demo device used for three-letter and nine-digit information encryption. The single key represents one external stimulus, while two keys represent the dual-key logic operation requiring both electric field and UV excitation simultaneously. Reproduced with permission from Ref. [96]. Copyright © 2017 The Royal Society of Chemistry.

Wang and co-workers developed RGB color-tunable “on-off” gel-type EFC devices (Table 1) [96]. The EFC mechanism involved using *p*-BQ as a redox-active electro-base, which, upon reduction, generates a strongly alkaline radical anion (Figure 6a). This in situ-generated base triggered the proton transfer-induced fluorescence activation of pre-selected pH-sensitive luminophores, namely 7-hydroxycoumarin (blue), rhodol (green), and 2-(2-(4-hydroxystyryl)-6-methyl-4H-pyran-4-ylidene)malononitrile (red). The solid-state RGB EFC devices featured a sandwich-type architecture comprising an EFC layer, a gel-type, ion-conductive layer based on PMMA, and an ion-storage layer (Figure 6b). High-fidelity fluorescence activation was achieved at a low voltage (−0.5 V), with sharp emission peaks at 457 nm, 539 nm, and 641 nm, respectively (Figure 6c). These devices exhibited fast switching speeds (*t*_on_ = 970 ms and *t*_off_ 760 ms for blue emission) and high CR (255 for blue, 156 for green, and 425 for red), along with an outstanding cycling stability (over 1000 cycles without performance degradation for B and G channels). Most remarkably, the RGB tunability enabled multiplexed encryption schemes. The device was designed such that both electric field and UV excitation (365 nm) must be applied simultaneously to reveal hidden emission patterns, fulfilling dual-key (and logic) operation requirements. Demonstration systems included a three-letter “SOS” pattern and a nine-digit digiboard capable of generating over 19,000 encryption combinations via color–position coding (Figure 6d). These features underscore the potential of electro-base-activated EFC platforms for high-capacity, secure, and reversible data encryption, where information remains fully concealed under either visual or electrical interrogation alone but becomes accessible only through the precise co-activation of external stimuli.

Other gel-type EFC platforms have also demonstrated significant promises for high-level encryption and anti-counterfeiting. Tang and co-workers developed dual-functional EFC AIE polymers capable of multilevel optical modulation under electrical stimulus (Table 1) [97]. By integrating electroactive TPA segments with aggregation-induced emissive cores, they constructed patterned devices that exhibited voltage-tunable fluorescence and color changes in both daylight and UV conditions. The EFC devices employed a sandwich-type architecture, in which the patterned electroactive polymers were deposited onto ITO glass substrates via a spray-printing method, and a gel electrolyte layer composed of acetonitrile, tetrabutylammonium perchlorate, propylene carbonate, and PMMA (in a 70:3:20:7 wt% ratio) was sandwiched between the polymer layer and a counter ITO electrode. These gel-based devices not only supported reversible dual-mode display but also enabled a four-dimensional color code encryption system, wherein spatial, color, emission, and electrical parameters were simultaneously encoded. Selective decryption required the correct combination of spatial position, applied voltage, and observation mode including UV excitation, thus implementing a “dual-key” authentication mechanism with excellent information density and security. He’s group reported a viologen-based EFC system incorporating AIE-active 9,10-azaboraphenanthrene (BNP) units, which exhibited reversible luminescence switching via a redox-controlled TICT mechanism [98]. The BNP-viologen compounds were weakly emissive in solutions but became highly fluorescent in the solid or aggregated state, making them ideal candidates for gel-phase EFC devices. The quasi-solid EFC devices showed reversible emission modulation enabling redox induced on/off switching with long-term cycling stability. Leveraging these properties, the authors successfully implemented a fluorescence-based encryption system, where the presence or absence of emissions corresponded to binary encoding. These systems highlight the capacity of AIE-active EFC gel-type devices to expand the chemical toolbox for secure information technologies.

### 4.3. Electronic Paper

**Figure 7:**
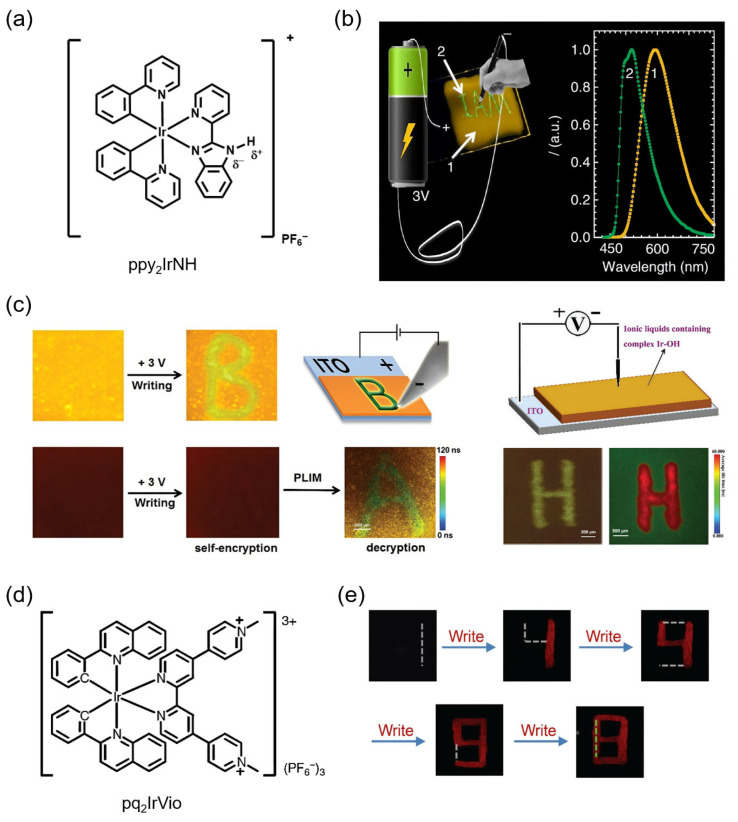
Ir(III) complex-embedded ionogel-type EFC devices for rewritable electronic paper. (**a**) Chemical structure of complex ppy_2_IrNH; (**b**) electronic paper under a UV lamp, and the emission spectra in gel-type device before and after applying a voltage. Reproduced from Ref. [99]. Licensed under CC BY 4.0; (**c**) voltage-induced writing of letters “A”, “B”, and “H” on gel-based electronic paper incorporating Ir(III) complexes bearing N-H or O-H moieties. Reproduced with permission from Refs. [100,101]. Copyright © 2014 WILEY-VCH Verlag GmbH & Co. KGaA, Weinheim. Copyright © 2016 WILEY-VCH Verlag GmbH & Co. KGaA, Weinheim; (**d**) chemical structure of complex pq_2_IrVio; and (**e**) photographs showing the stepwise writing and erasing of Arabic numerals for electronic paper containing pq_2_IrVio. Reproduced with permission from Ref. [102]. Copyright © 2016 WILEY-VCH Verlag GmbH & Co. KGaA, Weinheim.

The concept of electronic paper aims to replicate the readability and stability of traditional ink on paper while enabling rewritable and low-power content updates. Gel-type EFC platforms, combining soft-matter processability with redox-induced emissive control, offer unique opportunities for building flexible, rewritable, and high-resolution electronic paper systems. A representative demonstration by Zhao and co-workers reported a quasi-solid EFC device based on ionic Ir(III) complexes that simultaneously exhibited electrofluorochromism (Table 1) [99]. The molecular design involved an Ir(III) complex ppy_2_IrNH with a redox-responsive N-H moiety in its N^N ligand, paired with counterions such as PF_6_^−^, which formed tunable hydrogen bonds affecting the excited-state emission (Figure 7a). The quasi-solid EFC device was constructed using a composite gel matrix composed of BMIM-PF_6_ ionic liquid and silica nanoparticles, effectively forming a soft-solid electrolyte with excellent ion mobility and mechanical stability. Under a low voltage of 3 V, a platinum needle electrode was used to “write” patterns such as the word “IAM” on the gel film, locally triggering a green-to-yellow emission color change via electric field-induced ion migration (Figure 7b). The visual contrast and localized control enabled patterning without structural degradation, fulfilling the rewritable function of electronic paper. Unlike traditional paper relying on absorption modulation, this gel-based EFC system offered emissive switching with low driving voltages. In the follow-up studies, Zhao’s group further advanced this concept by designing a family of phosphorescent Ir(III) complexes with tunable EFC properties through ligand engineering [100,101]. By systematically varying the C^N ligands and employing N^N ligands bearing redox-responsive N-H or O-H moieties, the researchers developed a series of ionic Ir(III) complexes exhibiting emission colors ranging from green to red, which could be reversibly modulated upon electric field application. Electronic papers were fabricated by embedding the Ir(III) complexes into the previously used gel matrix composed of BMIM-PF_6_ and silica nanoparticles between an ITO substrate and a movable platinum tip electrode pen. When a voltage of 3 V was applied, distinct luminescent letters “A”, “B”, and “H” could be written onto the film by the local modulation of emission color or lifetime (Figure 7c).

Building on these efforts, the same research group explored alternative EFC mechanisms to further enrich the functionality of gel-based electronic paper. In another line of investigation, they developed a redox-active, transition-metal complex pq_2_IrVio bearing pendant pyridinium moieties [102] (Figure 7d). These electron-deficient groups acted as efficient quenchers of phosphorescence via PeT in the cationic state, while electrochemical reduction suppressed PeT and restored luminescence. Using a similar device configuration, the team constructed EFC devices based on these molecules with reversible write-erase capabilities. Upon applying a voltage of 1.5 V, local luminescence activation could be spatially controlled using a platinum probe as a “pen”, enabling the real-time writing of Arabic numerals on the film surface (Figure 7e). Reversal of the voltage polarity allowed for complete and rapid erasure using the same probe as an “eraser”. The write–erase cycles were repeatable and showed a <10% signal attenuation, demonstrating device robustness and high spatial fidelity in luminescence control. These works showcase the promise of gel-type EFC platforms in developing rewritable and low-power electronic paper technologies.

In a recent advancement, Hong et al. reported the development of soft EFC electronic paper using hydrogels doped with oligothiophene-conjugated benzothiazole derivatives, OCBs, which feature tunable solid-state emissions and excited-state intramolecular proton transfer behaviors (Table 1) [103]. The authors synthesized a series of OCBs with tailored conjugation lengths and terminal substituents, enabling emission tuning across the visible spectrum from blue to red. These fluorophores were stably incorporated into PVA hydrogel matrices through hydrogen bonding, yielding OCB@PVA composites with retained solid-state fluorescence and enhanced mechanical flexibility. The unique feature of this system lies in its electrochemically modulated emission response driven by pH changes within the hydrogel matrix. Upon the application of electric potential, the electrohydrolysis of water triggers either oxygen reduction or evolution reactions, leading to localized pH shifts that modulate the protonation state of the OCBs and thus their fluorescence output. Leveraging this mechanism, the team successfully constructed EFC devices based on OCB@PVA hydrogels in an electronic paper device. The OCB@PVA hydrogel with thickness ≈ 0.5 cm was placed on an ITO glass substrate and locally stimulated using a platinum “electronic pencil” connected to the cathode and operated at a low driving voltage of 2.4 V. Upon contact with the hydrogel surface, the tip of the pen induced a localized electrochemical reduction, triggering a visible fluorescence color change from green to orange, effectively enabling hand-written pattern formation. These patterns could be selectively erased or rewritten by reversing the polarity or repositioning the electrode, thereby achieving real-time and reversible writing/erasing functionalities. Moreover, the response time was significantly improved by pre-soaking the hydrogel in 0.1 M HCl solution for 10 s, which enhanced the proton availability and accelerated the fluorescence modulation. This user-interactive, low-voltage writing device demonstrates a practical and visually dynamic approach to gel-based EFC electronic paper.

### 4.4. Sensors

**Figure 8:**
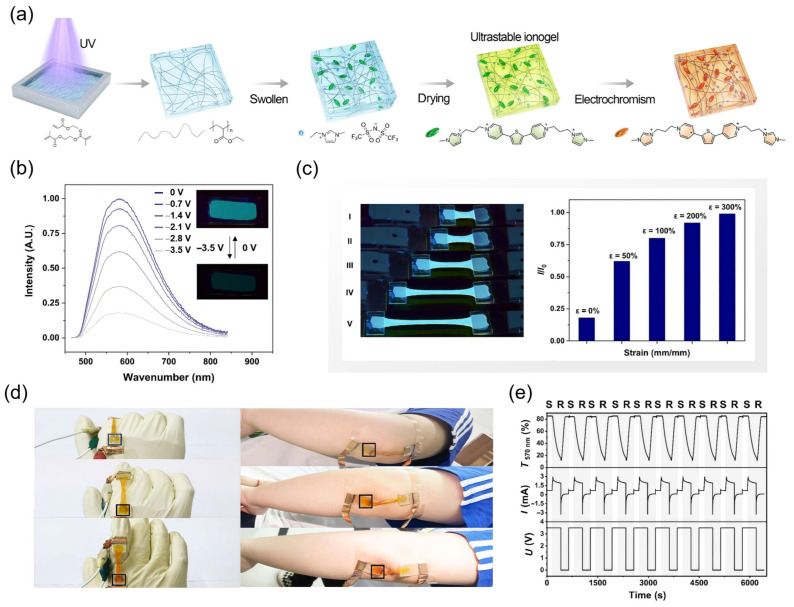
Stretchable viologen-based EFC ionogels for strain-responsive sensing. (**a**) Schematic illustration of the fabrication procedure of the [ImTV][TFSI]-containing ionogel; (**b**) electrofluorochromism of [ImTV][TFSI]-containing ionogel-based EFC device; (**c**) digital pictures of EFC behavior and fluorescence intensity of stretchable EFC device with [ImTV][TFSI] at different strain states; (**d**) digital pictures of stretchable device mounted onto the surface of a prosthetic finger and onto the elbow in stretched and relaxed states; and (**e**) transmittance at 570 nm and current versus time at stretched (S) and relaxed (R) states and alternating voltage of −3.5 and 0 V. Reproduced with permission from Ref. [41]. Copyright © 2022 Chinese Chemical Society.

Gel-type EFC devices also show great promise in developing visible and flexible sensors. Zhang and co-workers reported a multifunctional strain-sensing platform based on viologen ionic liquid-based ionogels, integrating EFC and strain-sensing functionalities within a single device architecture (Table 1) [41]. The ionogels were fabricated by incorporating viologen derivatives [ImTV][TFSI] into a double-network poly(ethyl acrylate) (PEA) elastomer matrix doped with [EMIM][TFSI] (Figure 8a). These materials exhibited a high transparency, mechanical stretchability up to 1000%, and long-term stability even after two years of storage, making them ideal for wearable sensors. Under applied voltage, the viologen units exhibited reversible EFC switching behavior, while the conductivity and optical response were simultaneously modulated by mechanical strain (Figure 8b). The relative fluorescent intensity of [ImTV][TFSI]-based devices increased from 1% to 99%, and with increasing strain increased from 0% to 300% (Figure 8c). This strain-dependent modulation is primarily attributed to the decreased ionic conductivity and impaired charge transport pathways within the ionogel matrix under tensile deformation, leading to a gradual suppression of EFC responses. To demonstrate practical utility, the ionogel-based EFC devices were conformally mounted onto dynamic substrates such as prosthetic fingers and elbows (Figure 8d). During mechanical deformation, such as bending or stretching, the devices exhibited a significant reduction or complete suppression in fluorescence and color intensity due to the strain-induced decrease in ionic conductivity (Figure 8e). Upon relaxation, the original optical response was restored, enabling reversible and visible monitoring of mechanical motion. This approach eliminates the need for external readout systems, providing an intuitive and energy-efficient platform for wearable optical strain sensing.

Gel-type EFC devices also offer unique advantages for biosensing applications by enabling the visual and low-voltage detection of biological targets with a high spatial resolution. A representative study by Tian and co-workers introduced a closed bipolar electrode-based EFC chip for the ultrasensitive and visual detection of cancer cell surface glycoprotein mucin-1 (MUC1) (Table 1) [40]. The device featured a dual-compartment architecture with anodic sensing and cathodic reporting cells separated on a PET substrate and confined within PDMS reservoirs, which provided a gel-like confinement for the electrolyte solutions. This quasi-gel configuration helped prevent leakage, stabilized ion transport, maintained compartmental integrity during operation, and provided good biocompatibility for sensing. In the anodic cell, MUC1 proteins or MCF-7 cancer cells were selectively captured using anti-MUC1 antibodies immobilized on chitosan–multiwalled carbon nanotube composites. Hybridization with ferrocene-labeled MUC1 aptamers enabled specific electrochemical recognition. Upon applying a driving voltage (3.5 V), ferrocene was oxidized at the anode, triggering the reduction in BQ to its radical anion BQ^•−^ in the cathodic chamber. This basic species activated the pH-sensitive fluorophore SPM, switching its emission from “off” to “on” and generating a visible fluorescence signal. The system achieved a limit of detection as low as 10 fM for the MUC1 protein and could detect as few as three MCF-7 cells. Importantly, the device maintained excellent specificity against interfering proteins and other cell types, while preserving the high viability of target cells under operational conditions. Confocal imaging revealed localized fluorescence activation only in response to the MUC1-positive cells, enabling the spatially resolved analysis of cellular expression. This work highlights the power of gel-supported closed bipolar electrode EFC systems in constructing integrated biosensors that convert redox biochemical interactions into intuitive optical signals. By combining modularity, sensitivity, and visual output, such platforms hold promises for portable clinical diagnostics, point-of-care screening, and single-cell analysis.

### 4.5. Multifunctional Devices

#### 4.5.1. Dual-Functional EFC Devices for Smart Windows and Displays

**Figure 9:**
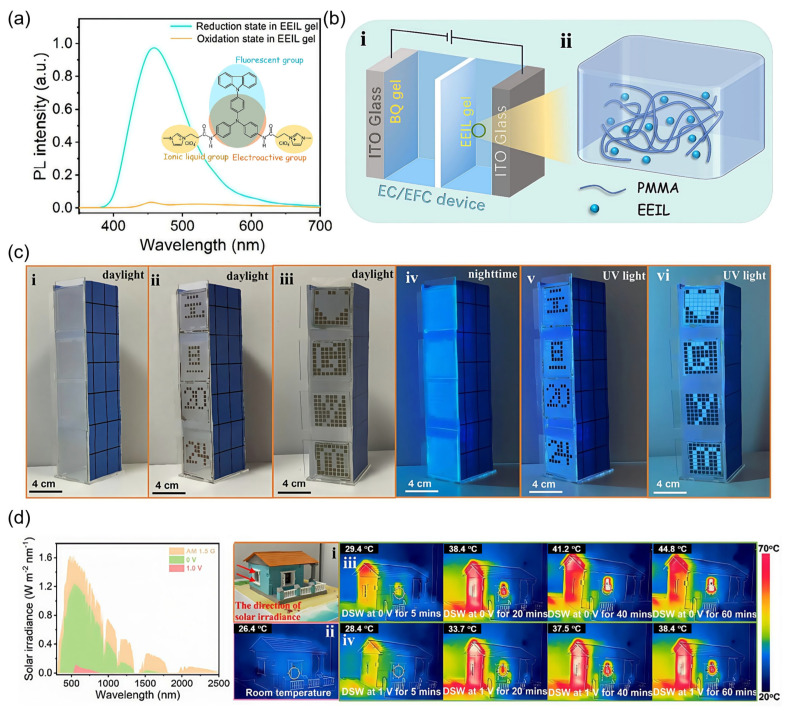
EEIL-based bilayer gel device for dual-modal smart window and EFC display applications. (**a**) Chemical structure and voltage-dependent modulation of aggregation-induced emission of EEIL; (**b**) schematic illustration of EEIL/PMMA-based bilayer gel device; (**c**) dual-modal display capabilities of the EEIL/PMMA-based device under different conditions: (i) daylight/no voltage; (ii, iii) daylight/with dot-matrix voltages; (iv) nighttime/no voltage; (v, vi) UV/with dot-matrix voltages; and (**d**) solar barrier effect of the device in the range of 300–2500 nm at 0 and 1 V, and infrared thermographic photos of the house model equipped with the EEIL/PMMA-based device at voltages of 0 and 1.0 V upon simulated sunlight exposure. (i) experimental setup of the house model; (ii–iv) thermal images under different conditions of room temperature, 0 V for 5 min and 1 V for 5 min. Reproduced with permission from Ref. [104]. Copyright © 2025 Wiley-VCH GmbH.

Among various multifunctional gel-type EFC platforms, devices that simultaneously serve as smart windows and EFC displays represent a particularly promising class of dual-functional systems. These integrated devices not only regulate solar transmission for energy efficiency but also provide dynamic visual output, addressing both practical and aesthetic demands in architectural applications. EC smart windows commonly dynamically modulate sunlight to reduce energy consumption for lighting, cooling, and heating. However, their lack of active display capability or nighttime visualization limits broader applicability. To overcome this constraint, Huang et al. developed a gel-type dual-functional EFC smart window that combines optical/thermal regulation with programmable reflective and emissive display capabilities (Table 1) [104]. By molecularly engineering an EFC ionic liquid EEIL incorporating TPA, carbazole, and imidazole moieties, the authors achieved an electroactive luminophore capable of voltage-dependent modulation of aggregation-induced emission (Figure 9a). To fully exploit this multifunctional behavior, a bilayer gel configuration was adopted, comprising an EEIL/PMMA-based emissive gel and a BQ-based ionic storage gel, both confined within a pixelated PET framework to allow for independent pixel actuation (Figure 9b). This configuration enabled low-voltage operation (<1 V), fast response times (<1.6 s), and high fluorescence CR (*I*_on_/*I*_off_ ≈ 1000). Notably, the device demonstrated dual-modal display capabilities across varying lighting conditions, rendering programmable visual patterns via EC reflection during the day and EFC fluorescence under UV illumination at night (Figure 9c). Simultaneously, it exhibited an excellent solar modulation efficiency, effectively blocking 99.3% of the solar heat in the NIR range (780–2500 nm) and obstructing the majority of the solar heat (97.9%) (Figure 9d). These features position this gel-type EFC device as a promising platform for smart windows that integrate energy-saving functionality with urban aesthetics.

Several other studies have also employed gel-type EFC devices to develop dual-functional materials involving smart windows. For instance, Navya et al. [105] designed heterocycle- and amine-free tetrabenzofluorene derivatives with excellent EC and EFC properties in gel-type devices (Table 1). While their proof-of-concept smart window primarily utilized the EC functionality for daylight modulation and energy saving, the intrinsic EFC behavior, evident in reversible fluorescence quenching upon oxidation, highlights the potential for nighttime emissive displays [105]. Similarly, Chang et al. developed a series of thienoviologen-based materials with extended π-conjugation bridges, which exhibited a strong NIR electrochromism and high CR in gel-type devices [106]. Their smart window prototypes were focused on EC effects, and the robust EFC performance of these viologen derivatives (*I*_on_/*I*_off_ up to 221) suggests great promise in dual-functional devices for smart windows and displays.

#### 4.5.2. EC/EFC Hybrid Display Systems

With the growing demand for multifunctional and energy-efficient display technologies, increasing attention has been devoted to hybrid systems that integrate both EC and EFC functionalities. These dual-mode platforms allow for a dynamic visual output under sunlight or UV excitation, making them attractive for applications. A number of gel-type EFC devices have been engineered to simultaneously realize EC and EFC behaviors within a single architecture, leveraging the soft-matter nature of gels to host multifunctional redox-active luminophores in mechanically flexible matrices [20,112,113,114,115].

Adams and co-workers developed a cost-effective, water-based chromogenic hydrogel display (CGD) by incorporating redox-active TTz derivatives into a PVA/borax hydrogel matrix (Table 1) [90]. These TTz molecules featured two distinct and reversible single-electron reductions, resulting in a three-state electrochromism: colorless/light yellow (TTz^2+^), purple radical cation (TTz^•−^), and blue neutral species (TTz^0^). Simultaneously, the neutral state exhibited strong photoluminescence, which was reversibly quenched (>90%) upon electrochemical reduction, allowing for the precise modulation of both absorption and emission within the same device. To achieve high reversibility and long-term cycling stability, the hydrogel matrix was doped with 1,1′-ferrocenedimethanol as a complementary redox mediator, promoting fast and reversible color and fluorescence switching at low voltages (<2.5 V). Notably, the (NPr)_2_TTz^4+^-based device retained over 94% of its optical contrast after 250 EC/EFC cycles, demonstrating exceptional operational durability. This work exemplifies the synergistic interplay between EC and EFC processes in gel-based soft electronics, providing a scalable strategy for EC/EFC multifunctional optoelectronic platforms.

**Figure 10:**
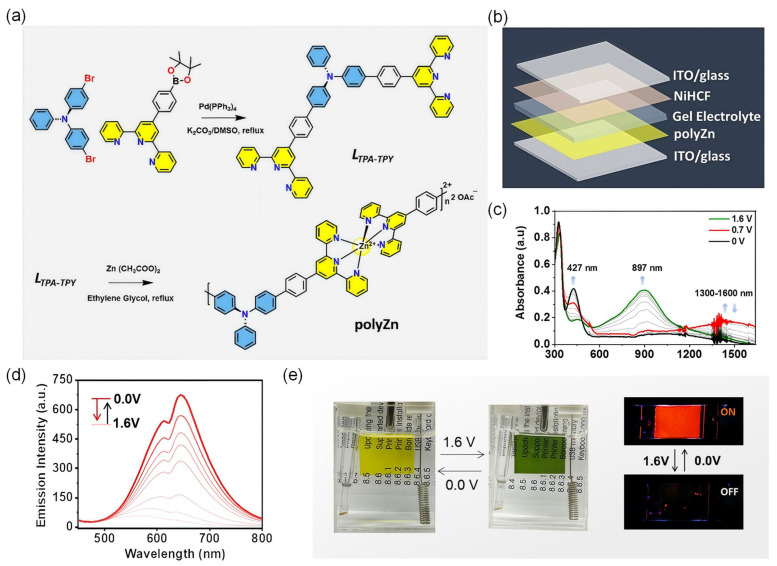
Redox-responsive metallo-supramolecular polymer enabling integrated EC and EFC performance. (**a**) Chemical structure and synthetic route of polyZn; (**b**) schematic presentation of the fabricated polyZn EC/EFC devices; (**c**) spectro-electrochemical absorption spectra of polyZn EC/EFC devices. The gray lines denote the spectral traces from 0.7 to 1.6 V, with arrows indicating the progression trend; (**d**) spectro-electrochemical emission spectra of polyZn EC/EFC devices; and (**e**) photograph of the redox-induced color and emission change in the polyZn EC/EFC devices. Reproduced with permission from Ref. [107]. Copyright © 2023 American Chemical Society.

Mondal et al. introduced a metallo-supramolecular polymer (polyZn) that integrates both EC and EFC functions into a single molecular system (Table 1) [107]. The polymer was synthesized via coordination of redox-active TPA derivatives with Zn(II) ions and terpyridine ligands, forming a linear coordination polymer with extended conjugation (Figure 10a). In its neutral state, the polyZn film exhibits a bright yellow color (*λ*_abs_ = 325 nm) and strong orange-red fluorescence (*λ*_em_ = 650 nm) under UV irradiation. The film was processed into solid-state EC/EFC devices using a sandwich configuration with a polymer gel electrolyte composed of LiClO_4_ and PMMA in propylene carbonate, providing mechanical stability and ionic conductivity (Figure 10b). Upon anodic oxidation at +1.6 V, the TPA units are oxidized to radical cations (TPA^•+^), resulting in the emergence of a strong NIR absorption band centered at 897 nm (Figure 10c) and dramatic fluorescence quenching up to 93% (Figure 10d), attributed to PeT from the excited Zn(II) center to oxidized TPA units. These changes are fully reversible upon reduction, with fluorescence and absorption properties restored nearly quantitatively, highlighting the robustness of the redox-triggered dual-mode modulation mechanism. The devices exhibited a visible color change between yellow and green and red luminescence switching (Figure 10e). Moreover, the cycling durability over 50 switching cycles with negligible performance loss, and the switching time for both EC and EFC transitions, remained within seconds, demonstrating the potential for practical applications. This work exemplifies how coordination chemistry and supramolecular design can be leveraged to achieve synergistic multifunctionality in gel-type EC/EFC platforms and paves the way to achieve unified EC/EFC behavior from a single polymeric backbone.

#### 4.5.3. Thermo-Responsive Systems with EFC Display

**Figure 11:**
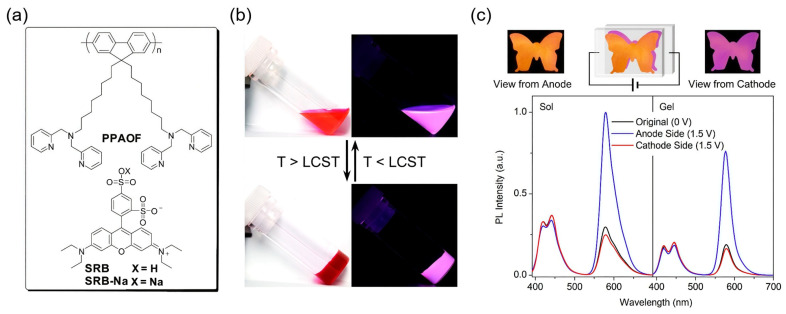
Dual-responsive EFC gel system with thermo- and electro-modulated fluorescence. (**a**) Chemical structures of PPAOF and SRB; (**b**) photographs of the thermo-responsive sol–gel transition of PPAOF-SRB. (**c**) Fluorescence images (excited at 365 nm) and photoluminescence spectra (excited at 380 nm) of the EFC device from anode and cathode sides under a potential of 1.5 V. Reproduced with permission from Ref. [108]. Copyright © 2017 American Chemical Society.

Thermo-responsive EFC systems with displays, which couple temperature sensitivity with voltage-dependent luminescence modulation, represent a promising avenue for multifunctional optoelectronic devices. Xiang and co-workers reported a representative example by constructing a supramolecular gel composed of dipicolylamine-functionalized polyfluorene (PPAOF) and sulforhodamine B (SRB) (Figure 11a) [108]. This material exhibits a lower critical solution temperature (LCST) at ~58 °C, enabling reversible thermal-phase separation. Below the LCST, PPAOF and SRB are well-dispersed, allowing for efficient FRET from blue-emissive PPAOF to SRB, yielding a dominant yellow emission (Figure 11b). Heating above the LCST disrupts this interaction, weakens FRET, and restores the blue emission of PPAOF (Figure 11b). This thermally regulated modulation enables ratiometric fluorescence as a visually distinct and reversible thermal indicator. In addition to its thermal responsiveness, the gel exhibits an excellent EFC performance. Electrochemical oxidation of PPAOF effectively quenches its fluorescence, and devices with asymmetric electrodes enable a regionally selective emission control. Blue emission can be locally suppressed under bias, while SRB fluorescence remains unaffected, producing spatially differentiated optical outputs (Figure 11c). The integration of a thermally tunable FRET pathway and electroactive fluorophore within a phase-changeable gel matrix allows for the precise modulation of emission color and intensity via dual stimuli (Figure 11c). This work underscores the potential of thermo-responsive EFC systems for smart thermal indicators and temperature-encoded encryption applications.

In another representative example, He and co-workers reported a gel-type EFC smart window based on poly(*N*-isopropylacrylamide-co-thienoviologen) copolymers (poly(NIPAM-co-TV)) [109]. By systematically varying the number of thiophene bridging units in the thienoviologen segments, the authors achieved tunable energy bandgaps (2.21–2.80 eV) and emission maxima (485–644 nm), along with high quantum yields (up to 92%) in the pristine state. Upon voltage application, the fluorescence emission was reversibly quenched due to redox-induced transformations of the viologen units. These copolymers were incorporated into quasi-solid EFC devices, in which aqueous solutions of poly(NIPAM-co-TV) were sandwiched between ITO electrodes to form thermally modulated emissive gels. Below the LCST at about 32 °C, the device remained transparent, while above the LCST, polymer chains collapsed into a hydrophobic scattering state, thereby triggering thermochromic transitions. Notably, under regional heating, fluorescence quenching was selectively induced in targeted areas, enabling the multi-mode optical control of the smart windows. The combination of thermal sensitivity, redox fluorescence modulation, and mechanical flexibility allowed these devices to function as smart windows capable of dynamic privacy control and visual pattern rendering. This work underscores the potential of gel-based EFC platforms in realizing smart windows that go beyond energy regulation to offer environment-adaptive display capabilities.

#### 4.5.4. Energy-Interactive EFC Display Devices

Energy-interactive EFC systems integrate energy harvesting or storage functionalities with optical modulation, enabling an autonomous or energy-efficient operation. These devices are typically categorized into two types: self-powered systems, which incorporate solar cells to drive EFC switching without external power input, and energy storing systems, which couple EFC modules with internal charge reservoirs such as batteries or capacitive layers. Gel-based materials play a crucial role in both configurations by providing ion-conductive, mechanically compliant, and multifunctional matrices that support energy conversion, storage, and fluorescence modulation in a unified platform.

**Figure 12:**
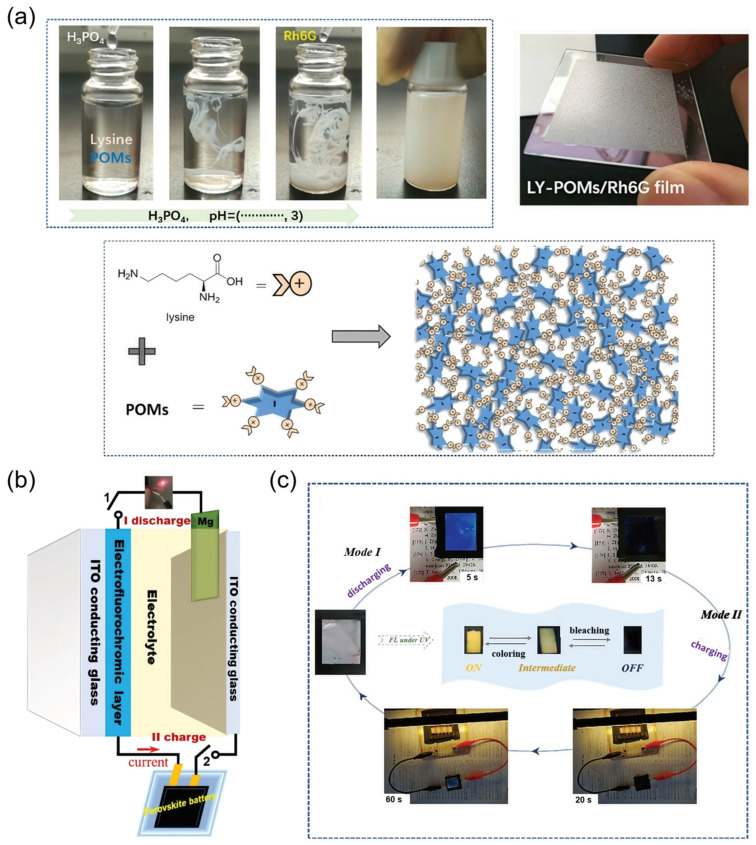
Self-powered gel-type EFC device based on supramolecular LY-POMs/Rh6G gel and integrated perovskite solar cell. (**a**) Co-assembly process of LY-POMs/Rh6G gels and chemical structures and packing model of LY-POMs and POMs; (**b**) schematic configuration of the discharging and charging processes of a self-powered EFCD; the arrow indicates the direction of the electric current; and (**c**) photographs of the reversible bleaching/coloration switching behavior of the self-powered EFC devices in the natural state (outside) and under UV (inset). Reproduced with permission from Ref. [110]. Copyright © 2019 The Royal Society of Chemistry.

A representative example of this concept was demonstrated by Sun and co-workers, who developed a fully self-powered gel-type EFC display device by integrating a perovskite solar cell (PSC) with a hybrid wet-adhesive gel electrolyte (Table 1) [110]. In this system, polyoxometalates (POMs) served as redox-active quenchers, while Rhodamine 6G (Rh6G) acted as the fluorescent emitter. These two components were co-assembled into a supramolecular gel matrix comprising lysine-functionalized POMs (LY-POMs) and silica-encapsulated Rh6G nanoparticles (Rh6G@SiO_2_), forming a multifunctional adhesive gel layer (Figure 12a). The device utilized a Mg/POMs redox pair to enable spontaneous discharge, while the PSC provided a photogenerated current for charge restoration, thus realizing a fully autonomous emission switching cycle without external power input (Figure 12b). Under UV illumination, fluorescence toggled between bright and dark states via a FRET-based mechanism from Rh6G to POMs, achieving a high CR of 0.8 with excellent reversibility over repeated cycles (Figure 12c). The gel matrix facilitated efficient ion migration and maintained the spatial organization of emissive components, contributing to stable and reproducible optical modulation. This work highlights a viable strategy for constructing self-powered, gel-based EFC display systems by coupling supramolecular photofunctional materials with integrated energy harvesting modules.

In addition to self-powered configurations, gel-type EFC devices have also been integrated with energy storage components to construct multifunctional supercapacitors. A representative example is the device developed by Zhuang and co-workers [111], which incorporated a triarylamine-functionalized viologen derivative (TPA-bpy) into a gel-based EFC–supercapacitor hybrid system. The molecular design of TPA-bpy featured a twisted TPA moiety linked to bipyridinium units, enabling the reversible multicolor switching between purple and yellow and electrofluorochromism upon voltage stimulus (Table 1). Importantly, TPA-bpy exhibited pronounced pseudocapacitive behavior with a wide voltage window (0–2.0 V), a high areal capacitance of 1.25 mF cm^−2^, and long discharge duration of 230.3 s at 0.01 mA cm^−2^. The charge–discharge processes were visually accompanied by optical transitions, offering the real-time monitoring of energy states through color change. The gel electrolyte, composed of PMMA, LiClO_4_, and propylene carbonate, provided a transparent and mechanically stable matrix for ion transport and redox modulation. Such integration holds great promise for flexible optoelectronics, particularly in applications requiring autonomous power management and dynamic visual feedback.

## 5. Conclusions

Gel-type EFC devices represent a promising frontier in the field of soft optoelectronics, offering unprecedented opportunities for combining electrical control, optical modulation, and mechanical flexibility within a single material platform. Through the rational integration of redox-active luminophores and tunable gel electrolytes, these systems address critical challenges associated with conventional EFC devices, including leakage, poor mechanical compliance, and limited multifunctionality. The diverse gel matrices not only serve as structural scaffolds and ion transport media but also play essential roles in stabilizing emission properties and enabling spatial pattern ability. Recent demonstrations of gel-type EFC systems across display, encryption, and sensing applications showcase their technological potential and versatility. Furthermore, the combination of EFC with electrochromism, thermochromism, and energy storage functionalities highlights the pathway toward smart, multifunctional devices that can dynamically adapt to environmental cues or user interactions.

## 6. Perspectives

Gel-type EFC devices have made significant strides in both fundamental materials design and device-level demonstrations. Key progress includes the development of multicolor, switchable luminophores; flexible, gel-based architectures compatible with wearable platforms; and improvements in switching speed and fluorescence contrast. Prototype devices have been successfully integrated into smart displays, anti-counterfeiting labels, sensors and electronic paper systems, showcasing the potential of EFC technology for next-generation optoelectronic applications.

Despite the impressive progress, several key challenges must be addressed to accelerate the practical application of gel-type EFC devices. First, the development of redox-stable luminophores with high fluorescence quantum yields and multi-color tunability remains a priority for achieving full-color and high-efficiency emission switching. Second, the design of gel matrices with simultaneously high ionic conductivity, mechanical resilience, and environmental stability is critical for wearable and implantable applications. Third, advanced fabrication methods such as 3D printing, screen printing, and inkjet deposition should be further explored to realize scalable and customizable EFC device manufacturing.

From the standpoint of future commercial applications, gel-type EFC displays and sensors still face additional challenges related to operational durability, packaging, and system-level integration. Most current demonstrations remain at the proof-of-concept stage, and transitioning to real-world products will require engineering efforts in device encapsulation, footprint minimization, and power management. For instance, wearable sensors and displays must be compatible with low-power electronics, thin-film batteries, or even self-powered configurations, while maintaining visual contrast and fast switching speed. In addition, the interfacing of EFC molecules with wireless systems, flexible substrates, or multiplexed circuits remains largely unexplored. Overcoming these integration barriers is essential for real-world deployment in sectors such as smart windows, wearable health monitors, and adaptive labels.

Looking ahead, future research should explore several directions:

(a) The molecular design of fluorophores with multi-state switching, environmental responsiveness, and improved fatigue resistance.
(b) The formulation of multifunctional gel matrices that combine ionic conduction, structural support, and protective encapsulation in a single component.
(c) The development of hybrid systems that combine EFC with other functionalities, such as energy storage, biosensing, or thermochromism.
(d) The creation of low-power, fully printed, or self-powered EFC circuits for flexible integration.
(e) The establishment of standardized protocols for evaluating long-term stability, optical contrast, and integration readiness.

In conclusion, gel-type EFC systems sit at the intersection of electrochemistry, materials science, and device engineering. Continued interdisciplinary efforts in molecular design, gel formulation, and device integration are essential for realizing their full potential in adaptive, intelligent, and energy-efficient optoelectronic technologies.

## Data Availability

No new data were created or analyzed in this study.

## Abbreviations

The following abbreviations are used in this manuscript:

AIE	Aggregation-Induced Emission
BQ	Benzoquinone
CR	Contrast Ratios
D-A	Donor-Acceptor
DFT	Density Functional Theory
EC	Electrochromic
EFC	Electrofluorochromic
FRET	Förster Resonance Energy Transfer
IEFs	Intrinsically Electroactive Fluorophores
ILs	Ionic Liquids
LCST	Lower Critical Solution Temperature
MD	Molecular Dynamics
NIR	Near-Infrared
PEA	Poly(ethyl acrylate)
PVA	Poly(vinyl alcohol)
PeT	Photoinduced Electron Transfer
PET	Polyethylene Terephthalate
PMMA	Poly(methyl methacrylate)
POMs	Polyoxometalates
TDDFT	Time-Dependent DFT
TICT	Twisted Intramolecular Charge-Transfer
TTz	Thiazolothiazole
TPA	Triphenylamine
TPE	Tetraphenylethylene
